# Clinical advancement of precision theranostics in prostate cancer

**DOI:** 10.3389/fonc.2023.1072510

**Published:** 2023-02-02

**Authors:** Yue Tan, Zhihui Fang, Yongxiang Tang, Kai Liu, Hong Zhao

**Affiliations:** ^1^ Hengyang Medical College, University of South China, Hengyang, Hunan, China; ^2^ Department of Nuclear Medicine, Xiangya Hospital, Central South University, Changsha, Hunan, China; ^3^ Key Laboratory of Biological Nanotechnology of National Health Commission, Xiangya Hospital, Central South University, Changsha, Hunan, China; ^4^ Department of Systems Medicine and Bioengineering, Houston Methodist Neal Cancer Center, Weill Cornell Medicine, Houston TX, United States; ^5^ Department of Gastrointestinal Surgery, The Third Xiangya Hospital of Central South University, Changsha, Hunan, China

**Keywords:** theranostics, imaging, prostate cancer, radiotracer, PET/CT

## Abstract

Theranostic approaches with positron emission tomography/computed tomography (PET/CT) or PET/magnetic resonance imaging (PET/MRI) molecular imaging probes are being implemented clinically in prostate cancer (PCa) diagnosis and imaging-guided precision surgery. This review article provides a comprehensive summary of the rapidly expanding list of molecular imaging probes in this field, including their applications in early diagnosis of primary prostate lesions; detection of lymph node, skeletal and visceral metastases in biochemical relapsed patients; and intraoperative guidance for tumor margin detection and nerve preservation. Although each imaging probe shows preferred efficacy in some applications and limitations in others, the exploration and research efforts in this field will eventually lead to improved precision theranostics of PCa.

## 1 Introduction

Prostate cancer (PCa) is the most frequently occurring cancer in men worldwide, with a continuously increasing incidence (1). Traditional methods for PCa diagnosis, including the digital rectal examination (DRE) and serum prostate-specific antigen (PSA) evaluation, cannot fully meet the diagnostic needs due to low accuracy and sensitivity (2). Novel methods, such as integrated positron emission tomography/computed tomography (PET/CT) or PET/magnetic resonance imaging (PET/MRI), to image ^68^Gallium(^68^Ga)-labeled prostate-specific membrane antigen (PSMA), which is exclusively overexpressed on clinical PCa cells, have brought great precision diagnostic capability. In addition to diagnosis, the major treatment strategy for PCa, prostatectomy, has entered the era of “precision surgery”, which requires a precise marking of the malignant tissue as intraoperative guidance. Identifying the actual position of the tumor, nerve, and lymph node has become more and more important during prostatectomy surgery. Novel intraoperative molecular imaging methods with high sensitivity, specificity, distinguishability, and safety, such as ^111^In labeled PSMA, have been shown to locate the PCa lesions precisely (3); indocyanine green (ICG), a USA Food and Drug Administration (FDA)-approved near-infrared (NIR) fluorescent agent for highlighting tissue, has been combined with ^99m^Tc to directly and accurately recognize malignant PCa tissue and metastases to assist decision making by surgeons during operations (4). In this article, we focus on providing a comprehensive summary of all novel molecular imaging probes in PCa diagnosis and intraoperative guidance for tumor detection and nerve preservation.

## 2 Novel Molecular imaging methods for PCa diagnosis

### 2.1 Prostate-specific membrane antigen

**Figure 1:**
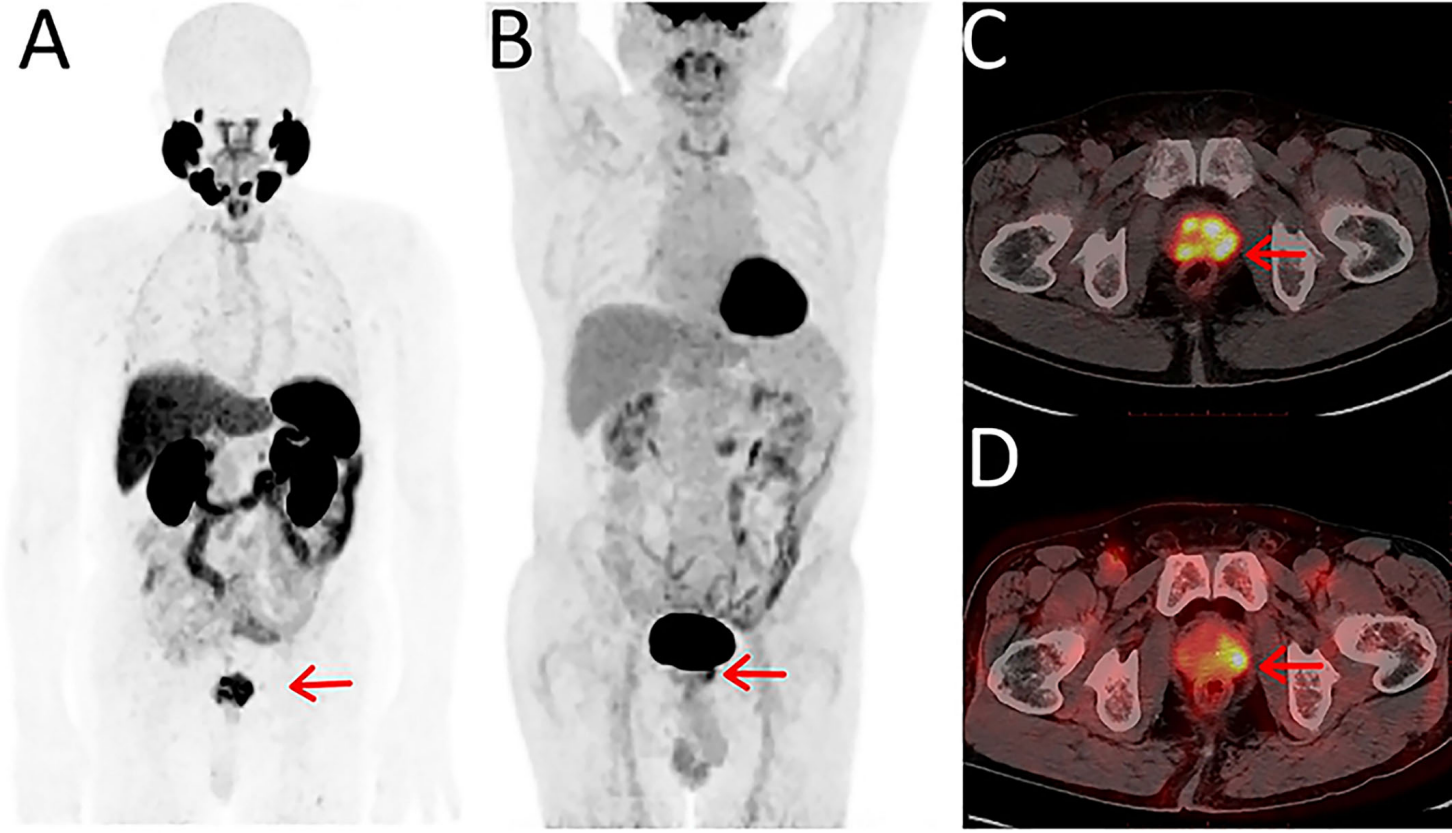
Maximum-intensity projections of PET examinations using ^18^F-PSMA-1007 **(A)** and ^18^F-FDG **(B)**. Axial PET/CT for ^18^F-PSMA-1007 **(C)** and ^18^F-FDG **(D)**. Reprinted with permission from Zhou et al. (9). Copyright ^©^ 2021 Zhou, Li, Jiang, Wang, Chen, Shen, You, Lu, Liao, Li and Cheng.

Prostate-specific membrane antigen (PSMA) is a type II transmembrane glycoprotein encoded by the folate hydrolase 1 (FOLH1) gene. Compared with other non-specific PET tracers, PET/CT imaging targeting PSMA has important clinical value in the diagnosis and staging of PCa. PSMA is highly expressed on the surface of PCa cells and is closely correlated with tumor grade, PSA value, and prognosis. So far, two PSMA agents (^68^Ga-PSMA11 and ^18^F DCFPyL) have been approved by the FDA for clinical application (5). Other PSMA tracers are also commonly used in preclinical studies and clinical trials, such as ^68^Ga-PSMA617 and ^18^F-PSMA-1007. A meta-analysis based on 37 studies with 4,790 patients was conducted by Perera et al. and showed that the overall sensitivity and specificity of ^68^Ga-PSMA PET/CT for initial staging of advanced PCa were 77% and 97%, respectively (6). According to a meta-analysis by Huang, an overall pooled detection rate of 94% for ^18^F-PSMA-1007 was demonstrated in PCa patients (7). With a combined median maximum standard uptake value (SUVmax) of 16 (3.7-77.7) for primary prostate lesions, ^18^F-PSMA-1007 had positive predictive values of 0.90, 0.94, and 0.84 with the identification of lesions, regional lymph node metastases, and localized prostate tumors, respectively. With the comparison of regular CT imaging and bone scanning, the accuracy of PET imaging with PSMA as the target was 27% higher (92% *vs* 65%), as were sensitivity and specificity (85% *vs* 38%, 98% *vs* 91%) (8). Zhou et al. made a critical comparison of ^18^F-PSMA-1007 PET/CT and ^18^F-FDG PET/CT, which were both performed on 21 PCa patients (9). The SUVmax, mean standard uptake value (SUVmean), and tumor-to-background ratio (TBR) of ^18^F-PSMA-1007 PET/CT were higher than those of ^18^F-FDG PET/CT in the primary lesions and metastases, leading to a superior detection rate in the primary PCa lesions and more significant differentiations between benign lesions and metastases. In addition, the multifocality of primary PCa lesions was presented under the ^18^F-PSMA-1007 PET/CT rather ^18^F-FDG PET/CT, suggesting the excellent PCa lession localization the PSMA tracer provides **(** **Figure 1** **)**.

PSMA PET/CT imaging makes a contribution to the diagnosis of PCa metastases. A prospective single-center study demonstrated that PSMA PET/CT imaging has modest sensitivity (71.4%) and exceptional specificity (88.9%) in detecting pelvic lymph node involvement (10). In addition, PSMA PET/CT imaging combined with sentinel lymph node biopsy in primary-identified medium to high-risk PCa resulted in 94% accuracy in original lymph node staging of PCa. This cross-validation could increase the overall sensitivity of lymph node metastasis to 100% (11). To assess the diagnostic efficacy of PSMA imaging for PCa bone metastases, a network meta-analysis that involved 45 studies with a total of 2,843 patients and 4,263 lesions was completed by Liu et al. (12). It evinced that ^68^Ga-PSMA PET/CT had an incredible ability to visualize bone metastases, with a superiority index of 7.3, which is higher than^18^F-NaF, ^11^C-choline, ^18^F-choline, ^18^F-fluorodeoxyglucose (FDG), and ^18^F-fluciclovir PET/CT. Harmon et al. compared the application of ^18^F-PSMA-PET/CT and ^18^F-NaF in bone metastases, and 185 bone lesions were recognized by ^18^F-NaF and/or ^18^F-PSMA in 26 patients, in which ^18^F-NaF significantly works better (p<0.001) (13). Van Damme et al. conducted a study on 134 PCa patients, including newly diagnosed and relapsing patients, to make a comparison between PSMA imaging and whole-body magnetic resonance imaging (WB-MRI) for metastases diagnosis (14). PSMA imaging and WB-MRI were found to have no significant difference among identifying PCa patients with metastases when lymph node, skeletal, and visceral metastases were considered as a whole. However, in the subgroup of newly diagnosed PCa patients, PSMA PET/CT was better than WB-MRI for the detection of lymph node metastases, suggesting PSMA PET/CT is superior to WB-MRI for the recognition of lymph node metastasis in early PCa.

Although there are superior advantages of PSMA PET-CT imaging ascribed to the high expression of PSMA in PCa, some limitations exist. High uptake of radionuclide has been found in some benign lesions and other non-PCa malignant tumors in clinical applications (15, 16). In addition, the efficacy of PSMA PET/CT imaging is highly susceptible to the PSA level. The PSA level was positively associated with the SUV value of ^18^F-PSMA imaging in patients with androgen deprivation therapy (ADT) (13). Combining PSA levels and treatment status, ADT patients (n=11) with a PSA below 2 ng/ml showed more lesions on ^18^F-NaF than on ^18^F-PSMA (p=0.02). Among patients with PSA > 2 ng/ml, ADT patients (n=8) showed the same or more lesions on ^18^F-PSMA than on ^18^F-NaF. In efficacy monitoring, ^18^F-PSMA-1007-PET/CT has a good localization function for the biochemical recurrence (BCR) of PCa patients with a detection rate of 75% (17). Even small lymph node metastases less than 8 mm in diameter were imaged clearly. However, the remaining 25% of patients with a low level of PSA were not recognized by ^18^F-PSMA imaging. Similar to ^18^F-PSMA PET/CT in the relationship between the detection rate of BCR and PSA level, ^68^Ga-PSMA PET/CT had this limitation in some studies (18, 19). Rauscher et al. analyzed subgroups of patients with very low (0.2-0.5 ng/ml) and low (0.5-1.0 ng/ml) PSA values and found that the detection rates of lesions in patients with recurred PCa were 55% (74/134) and 74% (102/138), respectively (18). Derlin et al. investigated the imaging efficacy of the ^68^Ga-PSMA PET/CT with more PSA level stratifications and presented much lower detection rates in patients with low PSA (< 2 ng/ml) (19). However, this limitation could be overlooked in castration-resistant prostate cancer (CRPC), which is characterized by a rising PSA. Fourquet et al. performed PSMA-PET/CT imaging in incomplete CRPC patients, which were defined as non-metastatic PCa patients after ADT treatment (20). Even for patients with PSA serum levels less than 2 ng/ml, the positive rate of PSMA PET/CT imaging could reach 70%, suggesting the high effectiveness of PSMA PET/CT imaging for CRPC-relevant diseases.

In addition, PSMA PET/CT imaging works well with the prognosis of PCa. Liu et al. used ^68^Ga-PSMA-617 PET/CT imaging semi-quantitative analysis indicators as “imaging markers” to predict risk stratification and metastasis risk of PCa (21). Univariate logistic regression models established by SUVmax, intraprostatic PSMA-derived tumor volume (iPSMA-TV), and intraprostatic total lesion PSMA (iTL-PSMA) could be able to efficiently previse high-risk PCa with the sensitivity and specificity of 87.5% and 50%, 62.5% and 100%, and 87.5% and 100%, respectively. A study performed by Roberts that included 848 patients after radical prostatectomy found that the SUVmax value of PSMA imaging lesions was remarkably negatively correlated with biochemical recurrence-free survival (BRFS) (22). Gleason score (GS) was also negatively correlated with BRF, and SUVmax value was an independent predictor of BRFS in patients. Roberts et al. found that increased ^68^Ga-PSMA-11 uptake is often associated with poor pathological outcomes and provides prognostic information for progression-free survival (23). Changes in PSMA expression could be a predictive biomarker for overall survival, which may assist in personalizing therapy for PCa patients (24). ^68^Ga-PSMA-11 PET/CT has a potential impact in guiding local lesion radiotherapy planning, which can improve the survival of castration-resistant PCa patients by adjusting the extent of radiotherapy (25). Under the guidance of PSMA PET, the mean time to PSA progression or last follow-up was 17.9 months with radiation therapy, compared with 2.9 months for patients without PSMA PET-guided local ablation radiation therapy (26). Shagera’s retrospective evaluation of 37 patients with metastatic hormone-sensitive or castration-resistant prostate cancer (mHSPC or mCRPC) by testing the biochemical association between responses and different PET parameters showed that ^68^Ga-PSMA-11 PET/CT imaging could be an effective tool for evaluating the response of metastatic PCa to taxane chemotherapy (27).

### 2.2 Neurotensin receptor 1

In addition to the specific molecular markers mentioned above, G protein-coupled neurotensin receptor (NTR) and its ligand neurotensin peptide (NT) have been suggested to play an important role in PCa. Inhibiting the pathway of NTR1 has been suggested as a possible strategy to prevent the pathogenesis of this disease (28). Morgat et al. performed a pilot study of the NTR1 expression in 12 samples of normal prostate, 11 samples of benign prostatic hyperplasia (BPH), 44 samples of PCa, and 15 samples of metastatic lymph nodes (29). Compared with the negative NTR1 staining in normal prostate and BPH samples, 4 of the 44 primary tumors (9.1%) and 5 of 15 metastatic lymph nodes (33.3%) overexpressed NTR1, suggesting that NTR 1 may be a potential biomarker of PCa, especially for metastatic lymph nodes. However, the limited sample series seriously affects the reliability of this conclusion, and a larger sample size is needed in future studies.

Nevertheless, studies have suggested that NTR1 may be another molecular target that could complement PSMA imaging. Ma et al. developed novel heterodimeric probes that targeted both PSMA and NTR1 and showed significant uptake in both NTR1-positive/PSMA-negative PC-3 tumors and PSMA-positive/NTR1-negative LnCap tumors (two androgen-sensitive PCa xenografts) at the animal level (30). Zhang et al. used ^68^Ga-DOTA-NT-20.3 animal PET imaging to scan the mice that were xenografted with PC-3, an androgen-receptor (AR)-positive, PCa cell line with no PSMA expression, suggesting that NTR1 may be a critical target for diagnosis or treatment of PCa applications with limited PSMA expression levels (31). However, the research of this tracer is still in the preclinical stage, and more preclinical and clinical studies are necessary for the exploration of its potential.

### 2.3 Fibroblast activation protein

First described as a cell surface antigen F19 in 1986, fibroblast activation protein (FAP) is a 760 amino acid (AA)-glycoprotein and a member of the dipeptidyl peptidase (DPP) family (32, 33). FAP shares a high AA sequence homology with DPP4, leading to its high DPP activity (34). In addition, endopeptidase activity for cleavage of benzyloxycarbonyl-glycine-proline-7-amino-4-methylcoumarin was also found in FAP (33). Just as it initially caught people’s attention for its existence in the mesenchyme of multiple cancers rather than epithelial cells, FAP was found to be overexpressed in most epithelial cancers and participate in the regulation of tumor growth and metastasis, suggesting that FAP is a potential target for tumor theranostic (35, 36). Currently, FAP inhibitors (FAPI) are mainly used for FAP-targeted PET/CT imaging (37). In 2018, Loktev et al. first reported that ^68^Ga-FAPI-02 was used for the imaging of multiple human malignant tumors and achieved good imaging results (38). Kratochwil et al. tested the ^68^Ga-FAPI-04 on 80 patients with 28 kinds of tumors, in which PCa patients showed intermediate uptake of ^68^Ga-FAPI-04 with SUV of 6-12 and TBR of 3-fold (39).

Kesch et al. developed tissue microarrays (TMAs) of prostate tissues from 94 PCa patients at various stages, including primary PCa, PCa receiving ADT, CRPC, and neuroendocrine prostate cancer (NEPC), with anti-FAP antibody staining, and found the positive correlation between FAP expression and disease advancement (40). The tissues with the highest FAP expression were from CRPC patients, suggesting the potential of FAPI imaging in advanced PCa, especially CRPC. A series of case studies for the FAPI PET/CT imaging on PSMA-negative CRPC also confirmed FAPI PET/CT imaging’s ability to visualize the metabolic lesions and complement the PSMA imaging (41–43). However, the issue of low sample size should be improved by large-scale clinical trials in the future.

The other weakness of FAPI PET/CT imaging is its specificity on tumor lesions. Xu et al. reported a case study of ^68^Ga-DOTA-FAPI-04 on a PCa patient with arthritis. Compared with the prostate lesions, the arthritis site presented higher uptake of FAPI, suggesting that ^68^Ga-DOTA-FAPI-04 may also be visualized in inflammation, possibly reducing its value in tumor diagnosis (44).

## 3 Additional PET agents for PCa diagnosis

### 3.1 ^18^F-fluorodeoxyglucose

For tumor PET imaging, ^18^F-fluorodeoxyglucose (^18^F-FDG) is one of the most frequently used radiotracers. Fluorodeoxyglucose (FDG) is a glucose analogue, which is highly absorbed in tumor lesions mainly through glucose transporter-1 (GLUT1) because of its involvement in tumor cell metabolism. It has been broadly applicated in clinical diagnosis, staging analysis, prognosis prediction, and treatment response monitoring of various tumors as a PET imaging agent (45). However, some patients with well-differentiated PCa had false negatives during clinical imaging (46). In addition, some benign lesions, such as inflammation, can also take up ^18^F-FDG. Since the prostate is close to the bladder and ^18^F-FDG is mostly egested through the urinary tract, this limits its application in the primary tumor of PCa due to the bladder urinalysis activity (9).

Although ^18^F-FDG imaging possesses limited accuracy on primary PCa diagnosis and staging, high-grade PCa (GS= 8-10) and more aggressive metastatic PCa showed higher glycolytic activity. In a study of 148 PCa patients with biopsy GS ≥ 8, ^18^F-FDG PET/CT imaging detected lesions with high intraprostatic FDG uptake in 66% of patients (47). Intraprostatic FDG uptake was positively correlated with higher pathological GS, seminal vesicle invasion, pathological lymph node metastasis, and risk of BCR, suggesting that preoperative intraprostatic FDG uptake is a composite factor for poor pathological prognostic factors. In addition, ^18^F-FDG has a certain value in the detection of primary lesions of CRPC. Chen et al. studied 56 cases of CRPC with ^68^Ga-PSMA and ^18^F-FDG PET/CT examinations (48). Although overall the ^68^Ga-PSMA is significantly better than ^18^F-FDG PET/CT with a higher detection rate of 75.0% *vs* 51.8%, and more positive lesions of 135 *vs* 95, the incidence of patients with ^68^Ga-PSMA−, ^18^F-FDG+ lesions was 23.2% (13/56), which could not be ignored in the clinic. The PSA level and GS of patients with ^68^Ga-PSMA−, ^18^F-FDG+ lesions were higher than those of patients without ^68^Ga-PSMA−, ^18^F-FDG+ lesions, that 61.5% of patients with GS ≥ 8 and PSA ≥ 7.9 ng/mL carried the special lesions, suggesting that CRPC patients with high GS and PSA may take advantage of ^18^F-FDG PET/CT imaging. ^18^F-FDG-PET/CT is also of great value in the diagnosis of bone metastases in high-grade PCa patients (GS≥8). In comparison with the bone scan, ^18^F-FDG PET/CT is sensitive and accurate in detecting bone metastases (sensitivity:100% vs 78.8%, specificity: 98.7% vs 98.2%) (49).

^18^F-FDG PET imaging also has the ability to assess prognosis in PCa patients. In the study of 94 patients with primary PCa who underwent ^18^F-FDG imaging previous to the radical prostatectomy, patients with higher SUVmax had poorer long-term survival (50). Higher intensity tracer uptake is positively associated with GLUT1 expression, stage, pathological grade, and disease progression. ^18^F-FDG PET whole-body total lesion glycolysis (TLG) is independently associated with overall survival as a quantitative prognostic imaging biomarker in mCRPC patients receiving abiraterone or enzalutamide as first-line therapy (51). Studies have shown that SUV value and the number of lesions are also independently associated with time to hormonal therapy failure (THTF). When the sum of SUVs was divided into quartile ranges, patients in the fourth quartile had significantly lower odds of survival than patients in the first quartile. Both SUV and ^18^F-FDG PET/CT-derived lesions provide independent prognostic information for THTF in patients with metastatic castration-sensitive PCa (52).

### 3.2 Choline

FDA approved the application of choline-based radiotracers (^11^C and ^18^F- choline) in 2012 for patients with biochemically relapsed PCa. Now both ^11^C and ^18^F- choline have been applied to monitor the curative effect in PCa patients. Wang et al. analyzed 46 studies and found that the combined sensitivity and specificity of ^18^F-choline for the detection of BCR of PCa were 0.93 (95% CI, 0.85-0.98) and 0.91 (95% CI, 0.73-0.97) (53). The combined detection rate was 66%, but when PSA is in the ranges of <0.5, 0.5-0.99, 1.0-1.99, and ≥2 ng/ml, the detection rates were 35%, 41%, 62%, 80%, respectively. Therefore, although the choline tracer is suitable for the detection of BCR of PCa, the detection rate is not ideal when the PSA value is very low.

**Figure 2:**
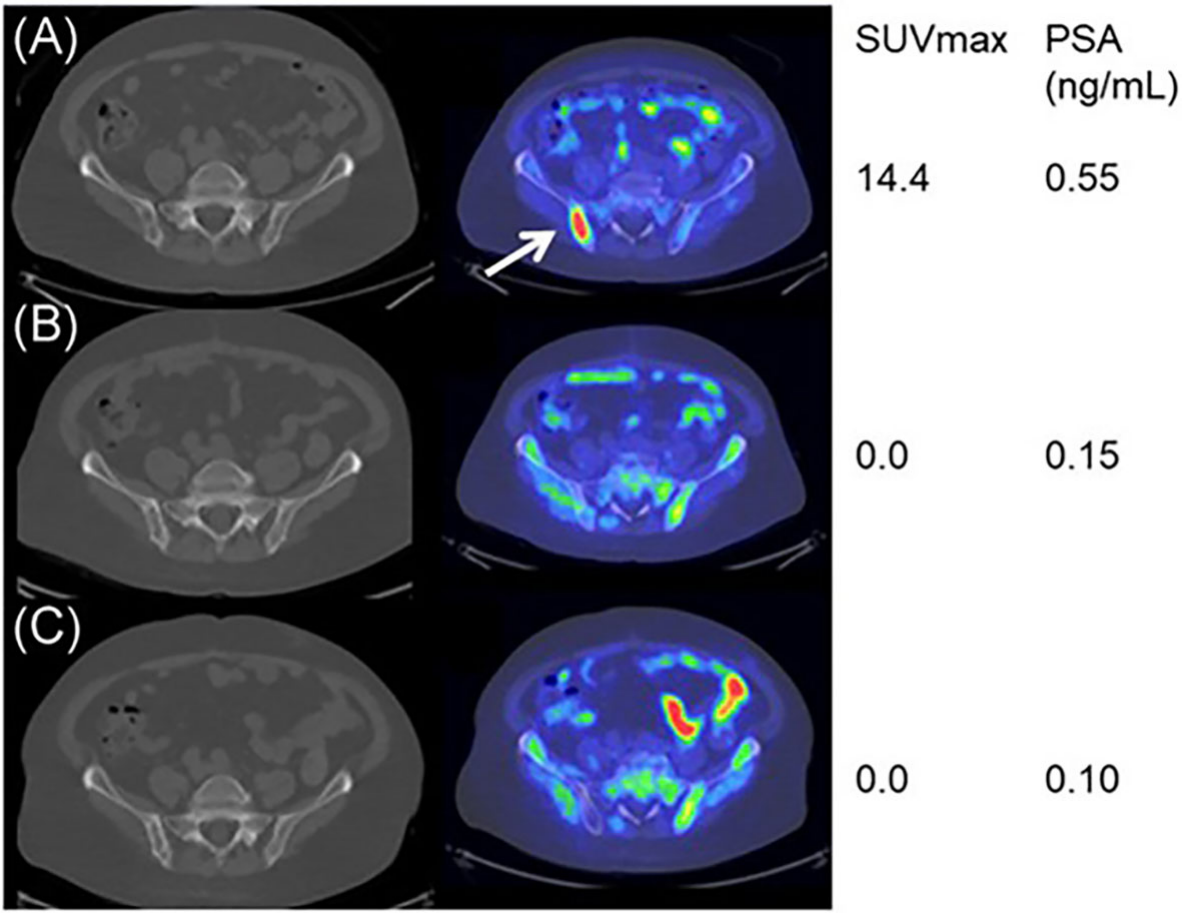
^11^C‐choline PET/CT imaging during the docetaxel chemotherapy for a good responder. Baseline **(A)**, mid‐course **(B)**, and posttherapy **(C)** axial fused ^11^C‐choline PET/CT images demonstrating markedly choline‐avid right posterior iliac bone metastasis at baseline (arrow), while nearly none at mid-course and posttherapy. Reprinted with permission from Jimbo et al. (54). Copyright ^©^2021 Wiley Periodicals LLC.

^11^C and ^18^F- choline also have implications in assessing prognosis in PCa. Jimbo et al. showed that ^11^C-choline PET/CT assessment in mCRPC patients receiving primary docetaxel chemotherapy could predict overall treatment response and progression-free survival with blood pool-corrected SUVmax during treatment **(** **Figure 2** **)** (54). The percent change in SUVmax was a significant predictor of complete response, with a greater than 20% reduction in SUVmax in 57 of 77 patients (74%), who were 3.6 times more likely to have complete remission than those patients with a reduction of SUVmax <20% after 6 cycles of primary docetaxel chemotherapy. Zhang et al. used ^11^C-choline-PET to identify 89 patients with oligometastatic CRPC, providing a better target for stereotactic ablative radiotherapy (SABR) to improve the outcome with a median overall survival of 29.3 months (55). García Vicente et al. conducted interim and end-of-treatment ^18^F-Fluorocholine (FCH) PET/CT imaging in ^223^Ra-treated CRPC and bone metastases patients, and the results were significantly associated with both progression-free survival and overall survival, suggesting that interim and end-of-treatment ^18^F-FCH PET/CT imaging could be applied as predictors and even guidance during the ^223^Ra therapy (56).

### 3.3 ^18^F- Fluciclovine

^18^F-fluciclovine (^18^F-FACBC) was first reported by Shoup in 1999 for brain tumor imaging (57). Based on the encouraging diagnostic presentation and histologically confirmed data in patients with biochemical recurrence PCa, the FDA and European Commission (EC) approved ^18^F-FACBC for diagnostic imaging in PCa patients with elevated PSA after pre-treatment (58), and until recently, ^18^F-FACBC imaging has been included in the National Comprehensive Cancer Network (NCCN) guidelines for the management of BCR of PCa. A previous phase II clinical trial found the sensitivity and specificity of the scan to be 92.5% and 90.1%, respectively, for primary PCa lesions (59). Uptake of ^18^F-FACBC was significantly increased in PCa primary lesions, and lesions with high GS (>3+4) tended to show higher uptake rates compared with low GS lesions and benign prostatic hyperplasia (60). In the diagnosis of lymph node metastases, this study found that only 1 in 7 patients with metastatic lymph nodes showed true positive results on ^18^F-FACBC PET/CT and PET/MRI. Another multicenter phase II study of 40 regional lymph nodes in 28 patients found that the sensitivity, specificity, diagnostic accuracy, positive predictive value, and negative predictive value of ^18^F-FACBC imaging in lymph node analysis were 57.1% (4/7), 84.8% (28/33), 80.0% (32/40), 44.4% (4/9) and 90.3% (28/31), respectively (61). ^18^F-FACBC PET/CT imaging has no advantage in the diagnosis of bone metastases either, possibly due to the low spatial resolution and partial volume effects caused by necrotic and mucinous components in the metastatic foci (62). A meta-analysis included 9 studies and found that the pooled sensitivity and specificity of ^18^F-FACBC imaging of aged PCa patients (including both primary and recurrent PCa) were 86.3% and 75.9%, respectively, with a combined diagnostic odds ratio of 16.453 and heterogeneity of 30% (63). In the regional analysis, ^18^F-FACBC-PET/CT owned a higher sensitivity and a lower specificity for the assessment of tumors in the prostate bed than in the extraprostatic region (90.4% *vs* 76.5%, 89% *vs* 45%, respectively). Filippi et al. studied the clinical data of 81 patients who underwent ^18^F-FACBC PET/CT for BCR of PCa (64). The detection rate of ^18^F-FACBC PET/CT in the entire cohort accounted for 76.9%, and the positive predictive value was 96.7%. This modality played an impact on the clinical management in 33 of 81 patients (40.7%), resulting in a critical amendment in treatment strategy in 30 subjects (90.9%). Like PSMA imaging, the detection rate of FACBC imaging is positively correlated with the PSA levels. When the PSA levels are in the range of 0.2-0.57, 0.58-0.99, 1-1.5 and >1.5 ng/ml, the detection rates of ^18^F-FACBC PET/CT were 66.7%, 71.4%, 78.9% and 90, respectively. However, even at a low PSA level, ^18^F-FACBC PET/CT imaging preserves a much higher detection rate than PSMA imaging, which is meaningful for the localization and diagnosis of lesions and has a significant impact on clinical management.

## 4 Other experimental radiotracers

### 4.1 Gastrin-releasing peptide receptor

Gastrin-releasing peptide receptor (GRPR) is a G protein-coupled receptor that is overexpressed in a variety of malignancies, such as breast cancer, PCa, and small cell lung cancer (65). GRPR is one of the subtypes of the bombesin (BBN) receptor, also called BB2r. As a bombesin analog, gastrin-releasing peptide (GRP) spreads over the peripheral nervous system and organs and primarily works in the gastrointestinal system through GRPR (66). As mentioned, the critical feature of GRPR is its overexpression in prostate tumor cells and underexpression in normal prostate tissue. Therefore, multiple radionuclides have been used to label bombesin analogs (GRPR agonists and antagonists), which preserve the high affinity for GRPR, to image tumors with high GRPR expressions (67, 68). At present, a variety of GRPR agonists and antagonists have emerged and been tagged with multiple radioisotopes. However, the GRPR agonists induce some gastrointestinal side effects due to the activation of GRPR. Compared with agonists, GRPR antagonists could provide better visualization with high value in the diagnosis and staging of PCa with less undesirable effects (69).

**Figure 3:**
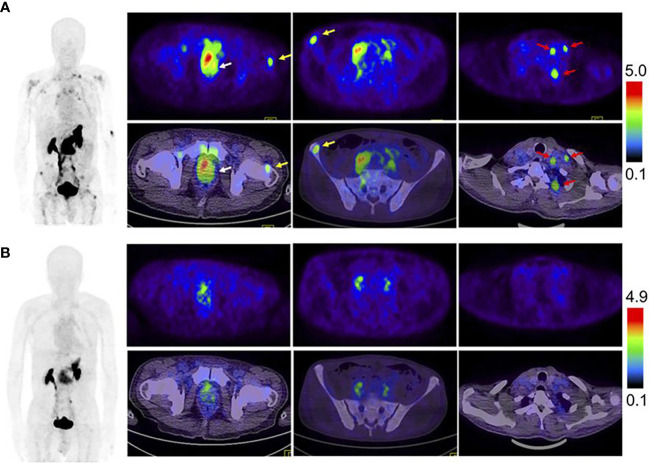
Comparison of ^68^Ga-RM26 PET/CT **(A)**, and ^68^Ga-BBN PET/CT **(B)** in a 73-y-old man diagnosed as having PCa (white arrow) with lymph node involvement (red arrow) and bone metastasis (yellow arrow) before prostatectomy. ^68^Ga-RM26 PET/CT detected primary tumors, multiple lymph node involvement, and bone metastasis lesion, whereas those lesions showed much lower uptake on ^68^Ga-BBN PET/CT. Reprinted with permission from Zhang et al. (70). Copyright ^©^ 2018 by the Society of Nuclear Medicine and Molecular Imaging.

As one of the GRPR antagonists, RM26 was radiolabeled to trace the GRPR in prostate tumor tissues. In Zhang’s study, both NOTA-RM26 and agonist BBN were labeled with ^68^Ga to image the lesions in 22 PCa patients (70). The results showed that the ^68^Ga-RM26 tracer visualized much more primary lesions and metastases with significantly higher SUVmax than ^68^Ga-BBN PET/CT **(** **Figure 3** **)**. Bakker et al. performed ^68^Ga-SB3 PET/CT imaging on 10 PCa patients before radical resection with a sensitivity of 88% and a specificity of 88% in 16 lesions detected by prostatectomy pathology, suggesting that ^68^Ga-SB3 PET/CT could be used for the detection and localization of primary PCa (71). Duan et al. compared ^68^Ga-RM2 PET imaging with multiparametric magnetic resonance imaging (mpMRI) and ^68^Ga-PSMA-11 PET on 41 patients with the initial diagnosis of intermediate and high-risk PCa. ^68^Ga-RM2 and ^68^Ga-PSMA11 had similar sensitivity and accuracy of 98%, 89% and 95%, 89%, respectively, which are significantly higher than mpMRI with 77% and 77%, for the detection of intraprostatic lesions (72). The post-prostatectomy histopathology also affirmed the ability of ^68^Ga-RM2 PET imaging with a detection rate of 93%.

Not only for the initial diagnosis of PCa, but GRPR-targeted PET imaging could also take a role in the follow-up with the detection of BCR. Minamimoto et al. conducted a prospective study of 32 patients with BRC of PCa but negative imaging results on multiple conventional imaging modalities (CT, MRI, and ^99m^Tc-MDP bone scan) (73). Among the 32 participants, 23 individuals were recognized through the ^68^Ga-RM2 PET imaging, suggesting a detection rate of 71.8% in these patients without positive findings on conventional imaging tools. Wieser et al. also collected 16 choline-PET/CT-negative/indeterminate biochemically recurrent PCa patients to evaluate the imaging ability of ^68^Ga-RM2-PET/CT in detecting metastatic tumors and found that tumors in 10 out of 16 patients (62.5%) could be recognized by the ^68^Ga-RM2-PET/CT imaging (74). In addition, the expression of GRPR appears to be unassociated with PSMA, suggesting that GRPR and PSMA-targeted PET imaging could be complementary (75). Therefore, GRPR-targeted imaging could complement other conventional modalities. Thus, GRPR tracer imaging is a promising tool for diagnosing and surveillance PCa with its high detection rate. However, the low sample size in these clinical trials critically affects the credibility of the evaluation of GRPR tracer imaging. More clinical trials with larger sample sizes are necessary for the future.

In conclusion, various imaging agents for the precision diagnosis of primary and metastatic PCa are under study, with both advantages and disadvantages **(** **Table 1** **)**. Although there is no 100% satisfactory imaging agent for PCa at present, with the in-depth research on current imaging agents and the development of new imaging agents, multi-target combined imaging or individualized imaging may bring better clinical value to PCa patients.

**Table 1:** Pros and cons of PET imaging agents for prostate cancer.

Tracers	Applications	Advantages	Limitations
PSMA	Diagnosis and staging	1. Excellent TBR; 2. High sensitivity and specificity to the primary tumor and lymph node metastases; 3. Combined with MRI has a good advantage in identifying CRPC; 4. FDA approved.	1. The detection of lesions is affected by the GS; 2. Imaging of non-metastatic CRPC patients is greatly affected by PSA level; 3. Sensitivity for the diagnosis of bone metastases is affected by PSA; 4. Positive on some benign lesions and non-PCa tumors.
	Curative effect monitoring and prognosis evaluation	1. A good localization function on recurrent PCa; 2. A valuable predictive biomarker for risk stratification and metastasis; 3. Be good at predicting overall survival and BRFS; 4. An excellent guidance for local lesion radiotherapy plan; 5. Works well in chemotherapy evaluation.	The detection rate for patients with BCR is affected by PSA level and GS.
NTR1	Preclinical studies	1. Clearly expressed in NEPC with low PSMA expression, that could be another molecular target that may complement PSMA imaging; 2. Works well on lymph node metastases.	A start-up tracer only for preclinical studies.
FAPI	Diagnosis and staging	1. Independent from blood glucose level, no need for prior rest, and fast image acquisition; 2. FAP imaging has the potential to guide the treatment of mCRPC; 3. Highly positive in patients with advanced CRPC	False positives due to the uptake of FAPI in benign lesions.
FDG	Diagnosis and staging	1. Wide range of clinical applications and easy to access; 2. High detection rate for CRPC.	1. False negatives with well-differentiated PCa; 2. False positive on some benign tissues; 3. Short half-life; 4. Low efficiency in primary PCa diagnosis; 5. Low sensitivity for lymph node metastases.
	Curative effect monitoring and prognosis evaluation	Preoperative intraprostatic FDG uptake is a good prognostic factor for poor pathology	
Choline	Diagnosis and staging	1. High specificity for the diagnosis of lymph node metastases; 2. More specific to bone metastases than bone scans.	1. Much affected by neoadjuvant ADT; 2. Low sensitivity to lymph node metastases (but better than traditional MRI); 3. Poor imaging of osteogenic bone metastases; 4. Less sensitive to bone metastases than bone scans
	Curative effect monitoring and prognosis evaluation	Well prognose mCRPC patients during chemotherapy or ^223^Ra treatment.	Not suitable for localization of lymph node metastases in patients with BCR, especially in the setting of low PSA values.
FACBC	Diagnosis and staging	1. The diagnosis of lymph node metastases is superior to choline PET imaging; 2. The overall sensitivity is higher in the prostate bed than in the extraprostatic region.	1. Limited role in local staging of PCa 2. False positive due to being non-specifically absorbed by benign prostatitis tissue; 3. The uptake was affected by GS; 4. Not suitable for bone metastases.
	Curative effect monitoring and prognosis evaluation	1. Little affected by clinicopathological parameters; 2. Works well for PCa restaging.	The localization and diagnosis of lesions in patients with BCR are affected by the level of PSA
GRPR	Diagnosis and staging	1. High detection sensitivity, specificity, and accuracy; 2. Independent of PSMA expression.	1. Low sensitivity to lymph node metastases; 2. Poor detection of bone metastases.
	Curative effect monitoring and prognosis evaluation	Lesion detection in patients with BCR of PCa is better than traditional imaging and choline PET/CT.	Affected by PSA growth rate.

## 5 Novel methods for intraoperative guidance of PCa precision surgery

Compared to diagnostic imaging tracers, there are fewer tracers available for guidance during PCa surgery. Herein, we present some novel intraoperative tracers, which are promising methods for PCa precision surgery in the future.

### 5.1 Novel methods for intraoperative tumor lesion tracing

Indocyanine green (ICG), one of the most common near-infrared (NIR) fluorophores for fluorescence-guided surgery (FGS), has been approved by the FDA for more than 60 years. It is a 776 Da, amphiphilic tricarbocyanine, water-soluble, and anionic probe. It binds to protein quickly and is confined to the intravascular compartment through intravenous injection (76). The half-life of ICG is 150-180 seconds, and it has low toxicity. Glutathione S-transferase, a transport protein, is able to make ICG through the liver and excrete into bile totally; thus, ICG can be administered repeatedly every 15 minutes during surgery to label the tissue (77). Due to its relatively low cost and widespread availability, ICG is widely used in urologic surgery, including laparoscopic and robotic adrenalectomy procedures (78, 79). In laparoscopic robot-assisted radical prostatectomy (RARP), Mangano et al. used ICG with NIR fluorescence to guide the preservation of the neurovascular bundle (80). Tobis et al. adopted ICG to highlight the renal vasculature and distinguish between normal and malignant tissue (81). Rho et al. used CT to guide the penetration of ICG through fluorescence thoracoscopy, precise location and margin resection of the radiopaque lesions were confirmed *via* C-arm fluoroscopy, and pulmonary nodules were resected with an endostapler (82). As a result, the ICG imaging guided pulmonary nodule removal was 100% in the 24 patients. However, due to the nature that ICG is a non-targeted probe with suboptimal emission characteristics for NIR-II detection, it cannot distinguish between benign and malignant tumors and can be accumulated by other tissues, which may cause false positives (83). This disadvantage was shown by Tummers et al. in a study on oncologic procedures of fluorescence-guided surgery with a high false-positive rate (62%) for the application of ICG (84).

As mentioned, PSMA is a type II integral membrane glycoprotein that shows elevated expression in the majority of PCa cells (85). It is a marvelous target for image-based intraoperative guidance for accurate tumor identification due to three reasons. First, PSMA is exclusively overexpressed on tumor cells of primary PCa lesions, while its expression is consistently low in healthy prostate tissues. Second, the expression level of PSMA correlates with the Gleason grading of PCa lesions. Last, binding with the extracellular domain of PSMA normally induces internalization of the imaging agents, resulting in substantial retention of the labeling inside the tumor lesions (86). PSMA radio-guided surgery (PSMA-RGS) has been approved to be an efficient method for resecting primary tumors and metastatic lymph nodes (87).

Intravenous injection of ^111^In-labeled PSMA-I&T to PCa patients during surgery has enabled the visualization of metastatic lymph nodes, which are normally unobtrusive and unrecognizable (88). Clinically, in patients undergoing salvage lymphadenectomy, the ^111^In-PSMA-RGS allows intraoperative detection of small lymph node metastases with high specificity and sensitivity (89). In addition, the ^111^In-PSMA-617 tracer also helped surgeons deal with unidentified pelvic lymph node metastases *in situ* during the surgery and resected ex vivo tissue samples to prove the successful removal (90). Except for ^111^In-labeled PSMA ligands for detecting metastases of PCa, Robu et al. explored another ligand named ^99m^Tc-mas3-y-nal-k(Sub-KuE) for PCa imaging (91). Clinically, ^99m^Tc is preferable to ^111^In, as it provides low-energy gamma rays that are more suitable for RGS due to the high sensitivity of gamma probes for collimation, and ^99m^Tc has a much shorter half-life (6 hours) than ^111^In (2.8 days), resulting in faster pharmacokinetics and lower radiation exposure for both patients and nuclear medical professionals (92).

The hybrid tracer ICG-^99m^Tc-nanocolloid combining fluorescent dye ICG with the radioactive ^99m^Tc-nanocolloid, not only offers preoperative sentinel node (SN) mapping, but also provides better optical surgical guidance (93). The tracer shows no leakage into the surgical field and provides a depth estimation (>0.5–1 cm) of the nodal location, which helps to prevent surgery-related side effects (94). Another study also approved the value of the hybrid tracer in the surgical identification of lymph nodes (95). Overall, one obvious advantage of the ICG-^99m^Tc-nanocolloid tracer is that it can enable visualization of any tumor lesion or SN in their anatomical context during surgery, and its application is independent of the order of resection (primary tumor or metastasis) or the surgical setting (open or laparoscopic) (94).

### 5.2 Novel methods for nerve protection in PCa surgery

**Figure 4:**
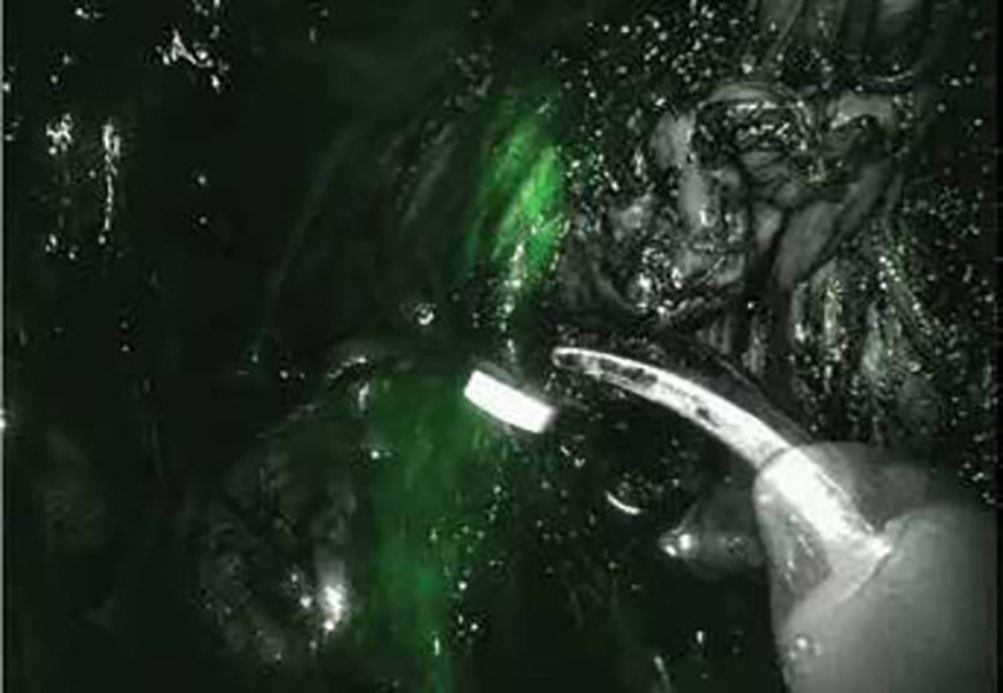
Prostate vascularization and neurovascular bundles by ICG. Reprinted with permission from Mangano et al. (80). Copyright ^©^ 2017 Wichtig Publishing.

**Figure 5:**
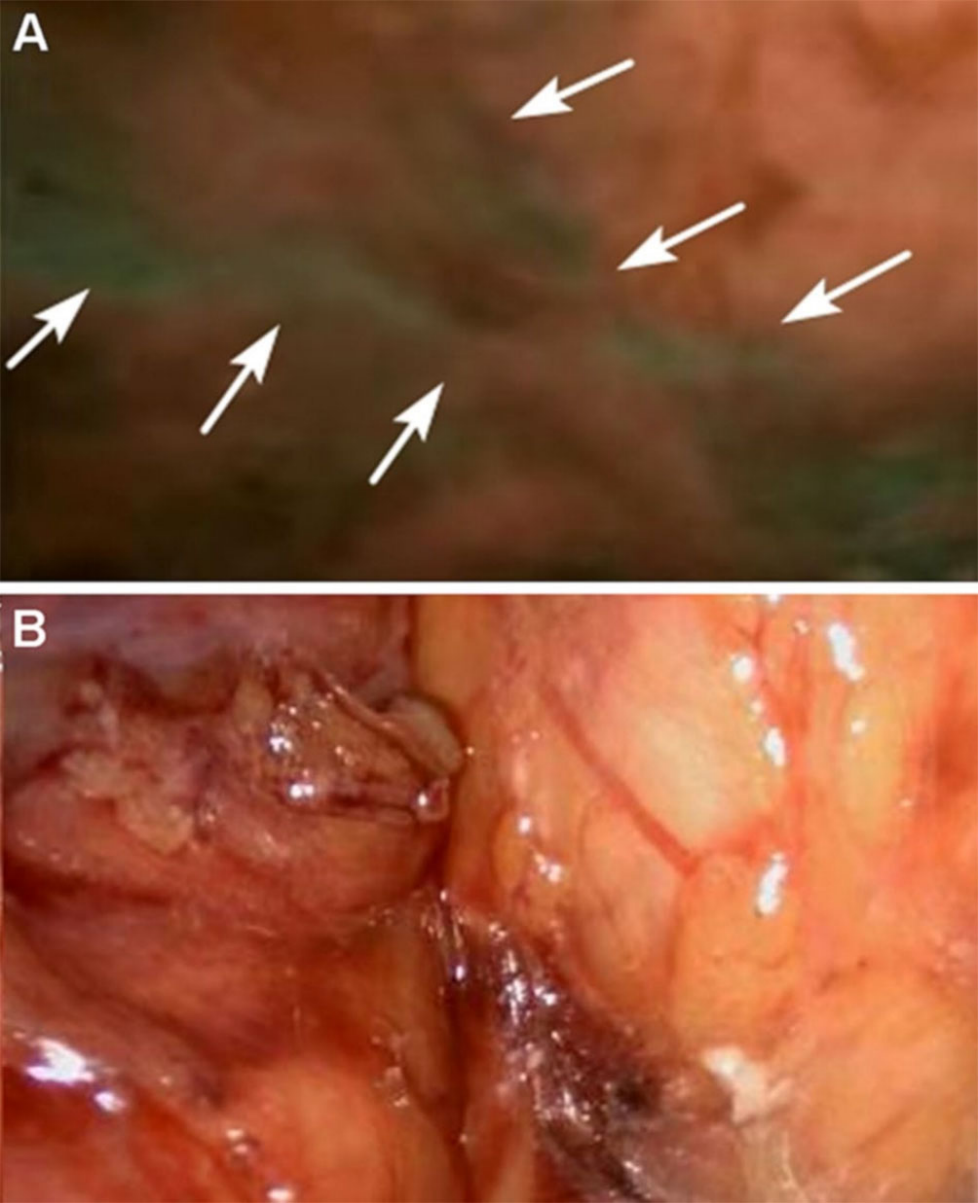
Autonomic pelvic nerves under the fuorescence **(A)** and under the white light **(B)**, the sacral plexus of autonomic pelvic nerves are displayed very clearly under NIR ray (white arrows) but not clearly under white light. Reprinted with permission from Jin et al. (100). Copyright ^©^The Author(s), under exclusive licence to Springer Science+Business Media, LLC, part of Springer Nature 2021.

Iatrogenic nerve injuries are common in prostatectomy, 20% of postoperative patients suffer from urinary incontinence, and many patients experience erectile dysfunction, which can only be partially mitigated by existing nerve-sparing surgical techniques. It is challenging to intraoperatively identify the specific location of buried small peripheral nerves (PNs), but the endeavor to find new ways to protect PNs is significant (96, 97). To meet the clinical need, an ideal method for imaging PNs during the intraoperative procedure should possess the following features. First, a high specificity and a good signal-to-noise ratio are essential. Second, real-time and long-term imaging is vital for PNs to be recognized and retained during surgery (98). Third, the imaging probes should have good biosafety. Last, the cost should be low enough for clinical use (97). Neurovascular dyes such as ICG and fluorescein have been used to highlight PNs in clinical settings (96). It has been shown that fluorescein was applied to visualize abnormal peroneal nerves in ganglion cyst excision procedures (99). Recently, ICG has been used to help protect critical functional structures in prostatectomy by enabling the identification of all neurovascular bundles without increasing the operative time or complications **(** **Figure 4** **)** (80). These promising data indicate that iatrogenic injury can be prevented, and the operative time can be shortened with the help of fluorescence-guided imaging. According to the clinical study performed by Jin et al., in patients with ICG injected 24 hours prior to surgery, the pelvic autonomic nerves can be intraoperatively seen clearly under a NIR ray **(** **Figure 5** **)** (100). Due to the ubiquity of such fluorophores, it is foreseeable that surgeons will attempt the fluorescent nerve-targeting agents more frequently in their clinical practice (96). However, the agents can have light penetrance through the tissue of greater than 5-6 mm. Such a deep penetration causes increased light scatter, thus obscuring the specific location of PNs. In addition, ICG is not a targeted dye, and it is not able to distinguish the nerve bundle from other tissues. For example, in the surgery for deep endometriosis, the ischemic lesion, the hypogastric nerve, the pelvic plexus, and the ureter were all dyed by ICG (101).

Extensive studies have reported that PCa tumor progression is favored by innervation. Magnon et al. reported that the formation of autonomic nerve fibers in the prostate gland regulates the development and dissemination of PCa (102). Therefore, biomarkers for innervation and effective visualization methods are necessary to assess nerve density in PCa. Nerve peptide 41 (NP41) has been found as a marker to highlight peripheral nerve tissue, and fluorescent-labeled NP41 can be visualized through its binding to the motor and sensory nerves in live mice (103, 104). Hingorani et al. reported that NP41 had the best nerve-to-non-nerve contrast compared to other peptides like NP38, 40, and 42, and the average nerve-to-non-nerve signal ratio increases by 17% under fluorescent imaging compared to white light (105). NP41 is considered an excellent agent for *in vivo* tracking of nerves in rodents. Since NP41 specifically targets nerves in PCa, it has the potential for visualizing nerve density and tumor innervation in PCa. The nanoprobes named propranolol-loaded-superparamagnetic iron oxide (SPIO)-NP41 nanoparticles (PSN NPs) have been used to assess the nerve density of PCa with high sensitivity and high specificity in mice (106). Since PSN NPs had an exclusive accumulation at the tumor site, benefiting the targeted delivery of propranolol, this study showed that PSN NPs inhibited PCa tumor growth by blocking the interaction between tumor cells and sympathetic nerves in the neural tumor microenvironment.

Nevertheless, existing data on applying NP41 to ex vivo human nerve tissue provided little contrast compared to muscle (105). Hence, human NP401 (HNP401), a peptide that binds to and highlights human autonomic and motor/sensory nerves, was identified for improving the labeling of human nerves, especially for the human prostate gland, suggesting its potential guidance role in the prostatectomy for PCa patients.

## 6 Expectation

Accurate and sensitive imaging using molecular probes is a promising and impactful method for early diagnosis of PCa. In addition, with molecular imaging-based intraoperative guidance, surgeons can achieve precise resection of the malignant PCa tumor as well as the metastatic lymph node, which is the trend in precision medicine. During prostatectomy, including robot-assisted radical prostatectomy (RARP), to maintain the function of the urinary system and erection postoperatively, fluorescent dye or labeled peptide hold great value in enabling visualization and protecting nerve bundles. Although each has disadvantages and limitations, all the novel methods discussed above are essential for developing early diagnosis and effective therapy of PCa. With endless exploration and research, more tracers with higher efficiency will appear to improve the precision theranostic of PCa.

## Author contributions

YT, KL and HZ conceived the theme. YT, ZF and YXT conducted the writing of the manuscript. YT and ZF prepared the figures and tables. KL and HZ edited and finalized this manuscript. All authors contributed to the article and approved the submitted version.

## Glossary

**Table:**

AA	Amino acid
ADT	Androgen deprivation therapy
AR	Androgen-receptor
BCR	Biochemical recurrence
BPH	Benign prostatic hyperplasia
BRFS	Biochemical recurrence-free survival
CRPC	Castration-resistant prostate cancer
DPP	Dipeptidyl peptidase
DRE	Digital rectal examination
FACBC	Fluciclovine
FAP	Fibroblast activation protein
FAPI	FAP inhibitors
FCH	Fluorocholine
FDA	Food and Drug Administration
FDG	Fluorodeoxyglucose
FGS	Fluorescence-guided surgery
FOLH1	Folate hydrolase 1
Ga	Gallium
GLUT1	Glucose transporter-1
GS	Gleason score
GRPR	Gastrin-releasing peptide receptor
HNP401	Human NP401
ICG	Indocyanine green
iPSMA-TV	Intraprostatic PSMA-derived tumor volume
iTL-PSMA	Intraprostatic total lesion PSMA
mHSPC or mCRPC	Metastatic hormone-sensitive or castration-resistant prostate cancer
mpMRI	Multiparametric magnetic resonance imaging
SUVmax	Maximum standard uptake value
SUVmean	Mean standard uptake value
NCCN	National Comprehensive Cancer Network
NEPC	Neuroendocrine prostate cancer
NIR	Near-infrared
NP41	Nerve peptide 41
NPs	Nanoparticles
NT	Neurotensin peptide
NTR	Neurotensin receptor
PCa	Prostate cancer
PET/CT	Positron emission tomography/computed tomography
PET/MRI	PET/magnetic resonance imaging
PNs	Peripheral nerves
PSA	Prostate-specific antigen
PSMA	Prostate-specific membrane antigen
PSMA-RGS	PSMA radio-guided surgery
RARP	Robot-assisted radical prostatectomy
SABR	Stereotactic ablative radiotherapy
SN	Sentinel node
SPIO	Superparamagnetic iron oxide
TBR	Tumor-to-background ratio
TLG	Total lesion glycolysis
THTF	Time to hormonal therapy failure
TMAs	Tissue microarrays
WB-MRI	Whole-body magnetic resonance imaging
